# Generating revenue by raising tobacco taxes in Latin America and the Caribbean

**DOI:** 10.26633/RPSP.2017.151

**Published:** 2017-12-12

**Authors:** Mark Goodchild, Rosa Carolina Sandoval, Itziar Belausteguigoitia

**Affiliations:** 1 World Health Organization World Health Organization Geneva Switzerland World Health Organization, Geneva, Switzerland.; 2 Pan American Health Organization Pan American Health Organization Washington, D.C. United States Pan American Health Organization, Washington, D.C., United States.

**Keywords:** Economics, taxes, public health, Latin America, Caribbean region, Economía, impuestos, salud pública, América Latina, región del Caribe, Economia, impostos, saúde pública, América Latina, região do Caribe

## Abstract

**Objective.:**

The objective of this study was to determine if raising tobacco taxes in the Latin America and Caribbean (LAC) region will generate extra tax revenue, even at outer edges of the sensitivity analysis, with relatively high price elasticities of demand for cigarettes.

**Methods.:**

A model of the cigarette market in 31 LAC countries was developed using cigarette tax, price, and retail sales data for 2014. It was then assumed that all countries increased the excise tax by 50% per pack. The model incorporated 12 studies from the LAC region that estimate the price elasticity of demand for cigarettes to quantify the expected impact of this tax increase on sales and tax revenues.

**Results.:**

The tax increase would raise cigarette prices by an average of 28% across the region. The volume of cigarette sales would decrease by 7% (confidence interval (CI): 3–11). Cigarette tax revenue would increase by 32% (CI: 27–37), representing an extra US$ 7 050 million (CI: 5 984–8 086) in revenue. Almost all countries showed increases in tax revenue, even at outer edges of the sensitivity analysis.

**Conclusions.:**

These findings confirm that the expected benefits of raising tobacco taxes are robust across the LAC region. Countries in the region should have confidence that raising tobacco tax rates will generate extra tax revenues.

## RESUMEN

**Objetivo.:**

El objetivo de este estudio fue determinar si aumentar los impuestos al tabaco en América Latina y el Caribe generaría ingresos fiscales adicionales, incluso por fuera del extremo superior del análisis de sensibilidad, con una elasticidad relativamente alta de la demanda de cigarrillos con respecto al precio.

**Métodos.:**

Se elaboró un modelo del mercado del cigarrillo en 31 países de América Latina y el Caribe utilizando datos sobre impuestos, precio y ventas minoristas de cigarrillos correspondientes al 2014. Luego se adoptó el supuesto de que todos los países aumentaban el impuesto al consumo de tabaco un 50% por paquete. El modelo incorporó 12 estudios de América Latina y el Caribe que estimaron la elasticidad de la demanda de cigarrillos con respecto al precio para cuantificar la repercusión prevista de este aumento de impuestos sobre las ventas y los ingresos fiscales.

**Resultados.:**

El aumento de los impuestos aumentaría el precio de los cigarrillos un 28% promedio en toda esta región. El volumen de la venta de cigarrillos disminuiría alrededor de 7% (intervalo de confianza [IC]: 3–11). Los ingresos derivados del impuesto a los cigarrillos aumentarían en 32% (IC: 27–37), lo que supondría ingresos adicionales por US$ 7 050 millones (IC: 5 984–8 086). En casi todos los países se observó un aumento de los ingresos fiscales, incluso por fuera del extremo superior del análisis de sensibilidad.

**Conclusiones.:**

Estos resultados confirman que los beneficios previstos del aumento de los impuestos al tabaco son sólidos en toda América Latina y el Caribe. Los países de esta región deben confiar en que el aumento de las tasas impositivas al tabaco generará ingresos fiscales adicionales.

## RESUMO

**Objetivo.:**

Determinar se o aumento dos impostos que incidem sobre os cigarros na Região da América Latina e Caribe (ALC) gera receita tributária adicional, mesmo nas extremidades da análise de sensibilidade, com elasticidade do preço relativamente alta na demanda por cigarros.

**Métodos.:**

Um modelo do mercado de cigarros em 31 países da ALC foi desenvolvido com a inclusão de dados sobre imposto, preço e vendas a varejo de cigarros em 2014. Pressupôs-se que todos os países efetuaram um aumento do imposto especial de consumo em 50% por maço de cigarro. O modelo se baseou em 12 estudos da Região da ALC que estimaram a elasticidade do preço na demanda por cigarros para quantificar o impacto esperado do aumento do imposto na receita tributária e das vendas.

**Resultados.:**

O aumento do imposto resultaria em um aumento médio no preço dos cigarros de 28% em toda a Região. O volume de vendas de cigarros sofreria uma queda de 7% (intervalo de confiança (IC): 3–11). A receita do imposto dos cigarros teria um aumento de 32% (IC: 27–37), representando um adicional de US$ 7,05 bilhões (IC: 5,984–8,086) na receita. Quase todos os países demonstraram aumento na receita tributária, até mesmo nas extremidades da análise de sensibilidade.

**Conclusão.:**

Os resultados deste estudo confirmam que os benefícios esperados com o aumento dos impostos dos cigarros são consideráveis em toda a Região da ALC. Os países podem ter confiança de que aumentar as taxas tributárias dos cigarros gera receita tributária adicional.

Tobacco taxation is one of the core demand-reduction strategies outlined in the World Health Organization (WHO) Framework Convention on Tobacco Control (FCTC) (1, 2) and is widely recognized as the single most potent and cost-effective strategy for curbing the demand for tobacco (3–5). Tobacco taxation has been described as a win–win policy because raising tax rates on tobacco can generate extra revenue for governments while also reducing tobacco consumption and the associated costs of illness. These twin benefits have been highlighted in various forums, including the Third International Conference on Financing for Development (FfD) held in Addis Ababa, Ethiopia, in July 2015, which produced the Addis Ababa Action Agenda (“Addis Agenda”). The Addis Agenda recognizes that “price and tax measures on tobacco can be an effective and important means to reduce tobacco consumption and health care costs, and represent a revenue stream for financing development in many countries” (6).

Despite taxation being considered one of the most cost-effective interventions to reduce tobacco use, it remains largely under-utilized in the Americas (7). This is largely due to tobacco industry tactics to block, delay, and weaken tobacco control policies. In the case of fiscal policies, governments often refrain from taking action because of claims propagated by the industry that higher taxes will harm economies and decrease tax revenues (5). These claims often center on the threat of increased illicit trade, the so-called Laffer effect (i.e., decreased tax revenue), or similar arguments that aim to weaken governments’ confidence in the expected relationship between price, demand, and tax revenue.

The strength of tobacco taxation as a means of controlling tobacco use and generating tax revenue hinges most critically on the price elasticity of demand. In empirical studies throughout the world, the price elasticity of demand for cigarettes is consistently found to be relatively inelastic—meaning that the percentage change in demand is less than the percentage change in price (3, 8). The inelasticity of demand provides the economic foundation upon which an increase in tobacco tax will decrease consumption yet increase overall tax revenue. To estimate the magnitude of these Effect Sizes (ES) more accurately, models used to forecast the impact of higher tobacco taxes often employ sensitivity analyses using a range of price elasticity estimates.

This study aimed to determine if raising tobacco taxes in Latin America and Caribbean (LAC) countries will generate extra tax revenue, even at the outer edges of the sensitivity analysis, with relatively high price elasticities of demand for cigarettes.

## MATERIALS AND METHODS

The data and methodology for the analysis were primarily derived from a study published in the WHO Bulletin in 2016 by Goodchild et al. (9). Data on taxes and prices for a 20-cigarette pack of the most popular brand in each country for 2014 were sourced from the 2015 *WHO report on the global tobacco epidemic* (4). In this dataset, the amount of excise and other taxes on cigarettes was calculated in U.S. dollars (US$) based on each country’s tax system. The quantity of licit (i.e., tax-paid) cigarette retail sales in each country was calculated using data from two market survey companies—GlobalData and Euromonitor International (10, 11). The dataset included 31 countries from the LAC region.

The retail price that consumers pay for cigarettes can be broken down into tax plus the industry price net of taxes (i.e., costs and profit margins). The tax component will, in turn, depend on the kinds of taxes that each country levies on cigarettes, though most countries apply excise and value-added taxes (VATs). A few countries, such as Belize, and Antigua and Barbuda, levy import or special duties rather than excise taxes. This study took the amount of excise tax (or duty) paid per 20-cigarette pack in 2014 and increased it by 50% in order to demonstrate the potential impact of a tax increase.

The robust—but not sharp—50% increase in the excise tax was chosen based on cigarette taxes and prices for the region, although many countries have been raising taxes on tobacco in much larger increments. For example, in May 2016, Peru increased its cigarette excise tax by more than 150% (from 1.4 to 3.6 soles/pack), and in December 2016, Colombia raised its excise tax on cigarettes by about 100% (from 701 to 1 400 pesos/pack). Another reason for choosing the 50% increase scenario was that it was expected to increase the retail price of cigarettes overall (region-wide) by an average of about 30%, a level of increase comparable with “real world” experiences. For example, WHO data show that the retail price of cigarettes increased by at least 20% in one-half of the countries that raised cigarette taxes between 2012 and 2014 and by more than 40% in one-quarter of them (4).

The model assumed in each country that 1) the industry price (i.e., costs and profit margins) was constant in real terms and 2) the tax increase was fully reflected in the new retail price (i.e., the post-tax increase retail price). While cigarette manufacturers in any country may choose to absorb, or over-shift, some or all of a tax increase, depending on various factors outlined below, the ex ante assumption of full tax pass-through reflects a “middle-ground” approach (i.e., no absorption or over-shift), which is consistent with the regional perspective (9). It also allowed the analysis to focus on the critical relationship between price and consumption: the price elasticity of demand.

The extent to which higher cigarette taxes and prices affect sales volumes is fundamentally determined by the price elasticity of demand. For example, a price elasticity of –0.3 means that a 10% increase in the retail price of cigarettes will reduce cigarette consumption by 3%. Studies in high-income countries have found elasticities that range from –0.25 to –0.5, while studies in other countries have found elasticities ranging from –0.2 to –0.8 (8). Guindon et al. (2015) completed a systematic review of price elasticity studies in Latin America and found they are likely to be below –0.5, with pooled estimates providing an elasticity of –0.31 (CI: –0.24 to –0.39) (12).

Table 1 includes data from 1) nine studies selected for this research (12–20) based on their use of time series data to measure consumption and 2) three additional studies (with data for Colombia, El Salvador, and Peru) that have been published since the systematic review (21–23). About half of the 12 studies used retail sales data; the other studies created composites of consumption based on official trade and production statistics. The time series studies were selected because they are more likely to reflect the impact of higher prices on the licit market—and hence tax revenues. In other words, studies that use cross-sectional data from household or individual surveys tend to generate lower estimates of the price elasticity of demand in part because they can measure both licit and illicit consumption and therefore might overstate the potential increase in tax revenues (3).

The model incorporated the price elasticities for the 12 countries listed in Table 1 to calculate the impact of a 50% increase in the excise tax on cigarette retail sales volumes in all 31 countries included in the model. The 12 core countries shown in Table 1 accounted for close to 90% of cigarette retail sales in the region (10, 11). The pooled elasticity estimates (CI: –0.24 to –0.39) from Guindon et al. (2015) (12) were applied to other highand upper-middle-income countries in the region that do not have studies of their own. However, it seems likely that smokers in lower-middle-income countries may exhibit greater price sensitivity. For example, the studies from Bolivia, El Salvador, and Guatemala showed relatively high elasticities (–0.85, –0.93, and –0.74 respectively). Therefore, for other lower-middle-income countries without studies, the model used a simple average of the elasticities in those three countries (–0.84; CI: –0.46 to –1.22).

## RESULTS

In 2014, smokers across the LAC region purchased 10.6 billion packs (or 212 billion cigarettes) from the licit retail market. The vast majority were purchased in South America, reflecting the large population of the countries and relatively high rates of adult smoking in that subregion. Total cigarette excise revenue across the LAC region came to US$ 16.4 billion in 2014. Other taxes, such as VATs, added another US$ 5.7 billion in tax revenue, resulting in a total tax revenue of US$ 22.1 billion. 

**TABLE 1:** Research on price elasticity of demand for cigarettes, including study method, type of data, country, country income group, and price elasticity with confidence intervals (CIs), Latin America and the Caribbean, 2014

Author (year; reference no.)	Method	Data	Country	Income group	Price elasticity	CIs
Martinez et al. (2015) (13)	VECM^a^	Sales	Argentina	UMIC^b^	-0.15	-0.11 to-0.19
Alcaraz (006) (14)	2SLS^c^	Sales	Bolivia	LMIC^b^	-0.85	-0.04 to-1.66
Iglesias et al. (2007) (15)	OLS^e^	P&T	Brazil	UMIC	-0.27	-0.10 to-0.40
Debrott Sánchez (2006) (16)	GARCH^f^	Sales	Chile	HIC^g^	-0.22	-0.20 to-0.24
Maldonado et al. (2016) (21)	2SLS	Sales	Colombia	UMIC	-0.79	-0.09 to-1.48
Ramos-Carbajales et al. (2016) (22)	VECM	Sales	El Salvador	LMIC	-0.93	-0.74 to-1.12
Gutiérrez (2010)^h^(12)	OLS	Tax	Guatemala	LMIC	-0.74	-0.61 to-0.81
van Walbeek et al. (2005) (17)	OLS	P&T^i^	Jamaica	UMIC	-0.23	0.13 to-0.59
Olivera-Chávez et al. (2010) (18)	OLS	P&T	Mexico	UMIC	-0.14	0.04 to-0.32
Herrera Ballesteros (2013) (19)	OLS	Imports	Panama	UMIC	-0.63	0.02 to-1.28
Gonzalez-Rozada & Ramos-Carbajales (2016) (23)	OLS	P&T	Peru	UMIC	-0.69	-0.06 to-1.32
Ramos & Curti (2006) (20)	2SLS	Sales	Uruguay	HIC	-0.34	-0.15 to-0.53

***Source:***Various authors as cited.

a VECM: vector error correction model.

b UMIC: upper-middle-income country.

c 2SLS: two-stage least squares.

d 2SLS: two-stage least squares.

e OLS: ordinary least squares.

f GARCH: generalized autoregressive conditional heteroskedasticity.

g HIC: high-income country.

h Unpublished data cited in (12).

i P&T: production and trade.

Table 2 shows the projected impact of raising the excise tax by 50% per pack in the region. If all LAC countries were to raise excise taxes by 50% per pack, the weighted average retail price of cigarettes would increase an average of 28% across the region. Average cigarette retail prices would increase the least in the Caribbean subregion, where baseline prices (net of tax) are relatively high. Smokers would respond to the price increase by purchasing fewer cigarettes, with the overall volume of tax-paid cigarette sales projected to decline by 7% in 2014, or a total of 723 million fewer cigarette packs consumed across the region. The quantity of cigarette sales declined by around 7% across all subregions, but there was a great deal of country variation, with Colombia, El Salvador, Guatemala, and Panama showing decreases in excess of 15% in this scenario, and many of the Caribbean islands, plus Belize, Mexico, and Paraguay, showing sales decreases of less than 5%.

**TABLE 2:** Projected impact of increasing excise tax by 50% per cigarette pack (change in retail price per pack, sales volume, excise revenue, and total tax revenue): central estimates by subregion/region, Latin America and the Caribbean (LAC), 2014

Variable	The Caribbean	Central America	South America	LAC region
Excise tax (US$/pack)	
2014 baseline	1.4	1.5	1.6	1.5
Projection	2.2	2.3	2.3	2.3
% change	50	50	50	50
Retail price (US$/pack)	
2014 baseline	4.4	3.1	3.1	3.1
Projection	5.3	4.0	3.9	4.0
% change	19	30	27	28
Cigarette sales (millions of packs)	
2014 baseline	208	2 179	8 219	10 606
Projection	196	2 041	7 645	9 882
Change	-12	-138	-574	-723
% Change	-6	-6	-7	-7
Excise tax revenue (US$ millions)	
2014 baseline	300	3 353	12 752	16 405
Projection	423	4 771	17 532	22 726
Change	122	1 418	4 780	6 321
% change	41	42	37	39
Total tax revenue (US$ millions)	
2014 baseline	428	4 268	17 385	22 092
Projection	577	5 890	22 675	29 142
Change	138	1 622	5 290	7 050
% change	32	38	30	32

***Source:***Author estimates.

Under this scenario, total tax revenues from the sale of cigarettes is projected to increase by around 32%, representing an extra US$ 7 050 million in revenue for governments across the region. Central America had the highest percentage ofincrease in total cigarette tax revenues (38%) versus the 2014 baseline. There was a wide spread of revenue growth across countries, with Argentina, the Bahamas,and Mexico recording increases of about 40%. However, all 31 countries included in the model showed positive and robust tax revenue growth.

Table 3 shows the range of outcomes for cigarette sales volume and total tax revenue based on the price elasticity CIs described above. For example, a 50% increase in the cigarette excise tax per pack is projected to decrease overall cigarette sales volume in the LAC region by –7% (CI: –3 to –11). The variation in Effect Size (ES) was greatest for the Central America subregion (–6; CI: –1 to –12) and low-er-middle-income countries (–12; CI: –7 to –17).

**FIGURE 1:**
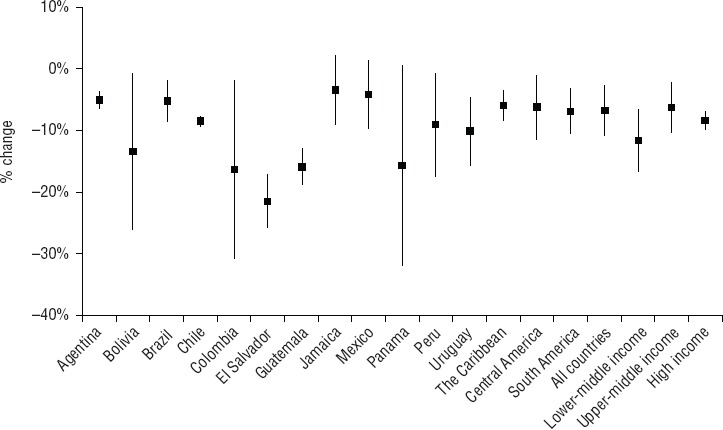
Projected change (%) in cigarette retail sales volume, by country, subregion/region, and country income group (lower-middle, upper-middle, and high), Latin America and the Caribbean, 2014

***Source:*** Author estimates.

Figure 1 shows the range in ES for cig-arette sales volume for 1) the 12 core countries listed in Table 1; 2) the three subregions (Caribbean, Central America, and South America); 3) the LAC region overall; and 4) the three country income groups (lower-middle, upper-middle, and high). The effects in Bolivia, Colombia, and Panama exhibited a relatively high degree of variation, with sales decreasing by more than 20% at the outer edges of the sensitivity analysis. The sensitivity analysis showed that cigarette sales could increase in some countries—Jamaica, Mexico, and Panama—even under the tax increase scenario. Cigarette sales volumes were projected to decrease by –6% (CI: –2 to –10) in upper-middle-income countries and by –8% (CI: –7 to –10) in high-income countries.

**TABLE 3:** Projected impact of increasing excise tax by 50% per cigarette pack (change in retail price per pack, sales volume, excise revenue, and total tax revenue): central estimates by subregion/region, Latin America and the Caribbean (LAC), 2014

Subregion/region and income group	Change in sales volume		Change in tax revenue	
	Millions of packs	%	US$ (millions)	%
The Caribbean	−7 to −18	−3 to −8	120 to 156	27 to 36
Central America	−24 to −252	−1 to −12	1 284 to 1 959	30 to 46
South America	−271 to −878	−3 to −11	4 580 to 5 971	26 to 34
LAC region	−303 to −1 147	−3 to −11	5 984 to 8 086	27 to 37
LMIC^a^ ( *n* = 7)	−42 to −105	−7 to −17	42 to 91	13 to 28
UMIC^b^ ( *n* = 17)	−204 to −960	−2 to −10	5 065 to 7 013	27 to 37
HIC^c^ ( *n* = 7)	−57 to −83	−7 to −10	878 to 983	33 to 37

***Source:*** Author estimates.

a LMIC = lower-middle-income country.

b UMIC = upper-middle-income country.

c HIC = high-income country.

**FIGURE 2:**
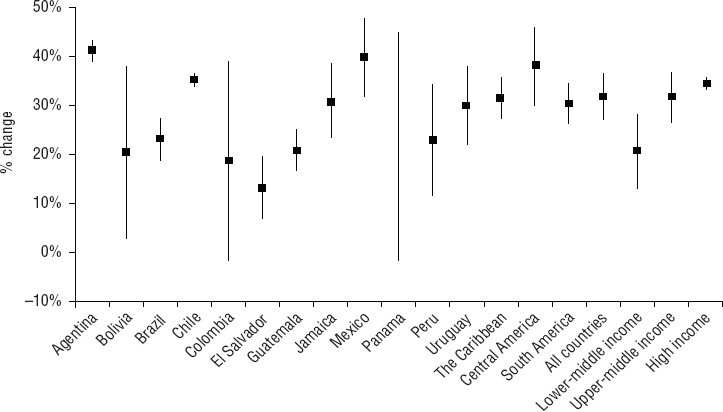
Projected change (%) in total cigarette tax revenue, by country, subregion/region, and country income group (lower-middle, upper-middle, and high), Latin America and the Caribbean, 2014

Figure 2 shows the range in ES for cigarette tax revenue for the same variables (the 12 countries, the three subregions, the LAC region overall, and the three country income groups). Under the tax-increase scenario, all 31 countries included in the model except two (Colombia and Panama) showed increased tax revenue at the outer edges of the sensitivity analysis (i.e., even under the most elastic price scenario). Although the median increase in total tax revenue for Colombia and Panama was around 20%, both countries had extremely wide CIs, causing the sensitivity analysis to be less definitive in these cases. More than half of the other 29 countries increased their revenue by 20% or more, even under the relatively high elasticity scenarios.

Raising the excise tax per cigarette pack by 50% in all of the LAC countries studied would increase overall cigarette tax revenue in the region by 32% (CI: 27–37), or US$ 7 050 million (CI: 5 984–8 086), which would help create the fiscal space needed by governments to finance their development priorities. If all of the extra revenue from the increased excise tax were allocated to public health budgets, government expenditure on health could increase by 2.5% region-wide (24, 25).

The sensitivity analyses showed that increasing the excise tax per cigarette pack by 50% would lead tax revenues in the lower-middle-income countries included in the model to increase by 21% (CI: 13–28). Total cigarette tax revenues in the upper-middle-income countries that were studied would increase by 32% (CI: 27–37), compared to 35% (CI: 33–37) in high-income countries. Central America tended to have the strongest performance, showing growth of 38% (CI: 30–46) in cigarette tax revenues.

## DISCUSSION

This study focused on cigarette retail sales in the licit market in order to assess the impact of higher tobacco taxes on cigarette tax revenues. To assess the impact of higher tobacco taxes on public health outcomes, however, the link between higher prices and the prevalence of smoking must also be considered. Because total cigarette consumption can include the illicit market, the impact of higher prices on smoking prevalence and total consumption could be weaker compared to just the retail market. In other words, researchers might expect the total (i.e., licit and illicit) demand for cigarettes to be relatively more price-inelastic.

***Source:***Author estimates.

Global evidence on the causal relationship between higher cigarette prices and the illicit market is mixed. This is especially the case with “large-scale smuggling”—a form of illicit trade that has the greatest potential to erode the tax base. In this part of the market, other factors, such as ineffective tax and customs administration, and the presence of criminal networks, are important determinants (3). On the regional level, data on the causal relationship between higher cigarette prices and the illicit market are scarce. Nonetheless, the experience in Brazil, where the reform process culminated in a significant increase in taxes on tobacco in 2011, is informative. Studies carried out there show that the reform increased tax revenue, and decreased overall smoking prevalence, despite an increase in the illicit market (26, 27): cigarette excise tax revenues more than doubled between 2006 and 2013, while tobacco use decreased from 34.4% to 14.7% of the adult population between 1989 and 2013 (26). Brazil’s experience supports the expectation that revenue and health objectives can be achieved even in the presence of smuggling.

Globally, the illicit market is present in low-tax jurisdictions as well as high-tax ones, which suggests it should be treated as a matter of risk management irrespective of the level of price and taxation. On the other hand, Ramos (2009) examined the dynamics of the illicit cigarette market in Argentina, Brazil, Paraguay, and Uruguay, and concluded that price was the main determinant of the illicit market in those countries (28). Nonetheless, experience from across the globe, including in middle-income countries such as Brazil, Kenya, and Turkey, confirms that measures such as tracking and tracing systems, licensing, stronger enforcement, and higher penalties can protect and enhance tax revenue collection (3).

The illicit tobacco trade is a transnational issue that requires greater regional cooperation and coordination, and the global community has a new instrument—the WHO FCTC Protocol to Eliminate Illicit Trade in Tobacco Products (“Illicit Trade Protocol” or ITP)—to help countries tackle it (29). Once in force, the ITP will require parties to implement a variety of control measures, with emphasis on those that strengthen control over the supply chain and improve cooperation. The ITP provides the blueprint for action against the illicit tobacco trade in the LAC region and worldwide.

### Limitations

There are a number of limitations to this study. First, the analysis did not delve into the exact tax policies needed by each country to raise its excise tax by 50%. Although there are a number of ways countries can achieve this increase, including global best practices outlined in the WHO FCTC Article 6 guidelines, such details are beyond the scope of this study (2). In addition, the analysis assumed there would be a complete pass-through of the tax increase by the tobacco industry in all countries, despite evidence that the extent of the pass-through can depend on a number of factors, such as the cigarette excise structure, the competitive dynamics of the cigarette market, and changes in the wider economic environment (30, 31).

For example, evidence from the European Union suggests that specific excise taxes (i.e., those based on quantity) have a greater impact on the retail price of cigarettes than ad valorem excise taxes (i.e., those based on value) (31). A recent study from South Africa found that the extent of under-shifting was significantly reduced by the entry of competitors into the low-price segment of the market (32). Other studies have highlighted the ability of monopolists to cross-subsidize by under-shifting the tax increase on low-price brands while over-shifting the tax increase on premium brands (33). The model’s middle-ground assumption of a complete pass-through precluded consideration of the different nuances of each market. However, it also allowed the analysis to focus on the price elasticity of cigarette demand—a determinant for which there is strong country-level evidence from across the LAC region.

### Conclusions

From a broader perspective, the findings of this study confirm that the expected benefits of raising tobacco tax rates are robust across the LAC region. Countries in the region should have confidence that higher tobacco tax rates will generate extra tax revenue and thus help create the fiscal space needed to finance development. The results for almost all of the 31 LAC countries studied showed solid increases in tax revenue from an increase in excise tax on cigarettes, even at the outer edges of the sensitivity analysis, with “high” price elasticities. The study also highlights that risk management measures to control the supply chain and improve cooperation among countries at the regional level will help protect and enhance the benefits of higher taxes from both the fiscal and public health perspective.

### Disclaimer

Authors hold sole responsibility for the views expressed in the manuscript, which may not necessarily reflect the opinion or policy of the RPSP/PAJPH, the Pan American Health Organization (PAHO), or the World Health Organization (WHO).
